# Supplementary material for: An evaluation of the Ultimatum Game as a measure of irritability and anger
Source: PLoS One. 2024 Aug 16;19(8):e0304038. doi: 10.1371/journal.pone.0304038 (PMC11329143; doi:10.1371/journal.pone.0304038)
Supplement: S1 File — (DOCX) [file pone.0304038.s001.docx]

S1 File. Supplementary Material on OSF (https://osf.io/gnj85/)
